# Effect of Yogurt and Its Components on the Deodorization of Raw and Fried Garlic Volatiles

**DOI:** 10.3390/molecules28155714

**Published:** 2023-07-28

**Authors:** Manpreet Kaur, Sheryl Barringer

**Affiliations:** Department of Food Science and Technology, The Ohio State University, Columbus, OH 43210, USA; kaur.333@osu.edu

**Keywords:** garlic, cooking, volatiles, deodorization, yogurt, fat, proteins, heating, pH

## Abstract

Garlic contains sulfur volatiles that cause a bad odor after consumption. The objective of this study was to understand how yogurt and its components cause deodorization. Raw and fried garlic samples were mixed with various treatments and measurements of volatiles were conducted using a selected-ion flow-tube mass spectrometer. Frying garlic significantly reduced almost all sulfur volatile compounds. Raw garlic was deodorized more than fried garlic by all of the treatments. Fat, protein and water significantly reduced the concentration of sulfur-based volatiles in garlic. At the same concentration, either fat or protein produced higher deodorization, depending on the hydrophobicity of the volatile. Whey protein, casein and their complex all caused deodorization. Increasing the pH to 7 or heating changed the structure of the proteins and decreased the deodorization of the volatiles, showing the importance of proteins for deodorization. As the quantity of fat increased, the deodorization of the volatiles also increased. Foods with higher fat or protein content can be formulated to offer a potential solution to reduce the unpleasant odor associated with garlic consumption.

## 1. Introduction

The use of garlic (*Allium sativum* L.) as a spice in the food industry is common [1,2]. Most of the characteristic volatiles in garlic contain sulfur [3]. In raw garlic, the primary sulfur-containing compound is alliin. When the garlic is crushed, chopped or otherwise disrupted, the enzyme alliinase converts alliin into allicin, which is responsible for the pungent aroma of fresh garlic. However, allicin is unstable and breaks down quickly to form allyl methyl disulfide and diallyl disulfide. Diallyl disulfide is reduced to form allyl mercaptan. Allyl mercaptan is methylated to form allyl methyl sulfide [4,5]. These volatiles are responsible for the malodorous nature of garlic [4,5]. Diallyl disulfide and allyl mercaptan are the volatiles with the highest concentration in garlic [5].

Various foods, such as vegetables, fruits and herbs, and beverages have been used to deodorize garlic volatiles. Treatments including mint leaves (spearmint and peppermint) [6,7], pear, loquat, peach, plum, prune, apricot, cherry, grape, chicory, udo, perilla, burdock, potato, eggplant [7], dried and fresh herbs [8], lemon juice, green tea [9], spinach, parsley [9,10], basil [7,10], kiwi [10], raw egg, boiled rice [10], mushrooms [7,10], apple [7,9,11], raw lettuce [7,10,11], milk [10,12] and oil [12] have been used to reduce the concentration of volatiles in garlic.

Several different mechanisms have been proposed for deodorization. The raw vegetables, fruits and herbs were proposed to cause deodorization due to their enzymatic and phenolic activity [6,8,9,11]. The enzyme polyphenol oxidase (PPO) catalyzes the oxidation of the sulfur compounds responsible for the odor of the garlic [6,8,9,11]. Milk deodorizes due to the water and fat present in it [10,12]. The milk fat deodorizes the garlic volatiles by creating a hydrophobic environment [12]. The water reduces garlic volatiles by partitioning volatiles between phases [12]. Proteins deodorize by the adsorption of volatiles through hydrogen and hydrophobic bonding [13,14]. The protein casein is proposed to interact through disulfide interactions or sulfhydryl bonds [12,13]. Whey protein interacts through hydrophobic interactions [13].

Yogurt is a fermented milk product that is popular among consumers for its health-promoting, nutritional and sensorial properties. Yogurt has 0.15–5% fat, 3–10% protein and 70–87% water depending on the type of yogurt [15]. However, yogurt has not been tested for its ability to reduce the concentration of the sulfur volatiles in garlic. Therefore, the objective of this study is to test yogurt and to understand the mechanism behind the deodorization of both raw and fried garlic with yogurt and its components.

## 2. Results and Discussion

### 2.1. Effect of Frying on Raw Garlic Volatiles

The volatiles responsible for most of the pungent odor in both raw and fried garlic are allyl methyl disulfide, diallyl disulfide, allyl mercaptan and allyl methyl sulfide [12,16]. Allyl methyl disulfide has a garlic- like, savory odor [17]. Diallyl disulfide has a pungent, garlic- like odor with an odor threshold of 0.22 ppb [17]. Allyl mercaptan has a sulfurous odor with an odor threshold of 0.05 ppb [18]. Allyl methyl sulfide has a garlic- like, savory odor with an odor threshold of 0.14 ppb [17]. All of these volatiles were above their threshold level in both raw and fried garlic, which means the pungent odor of these volatiles is perceived or detected by the human nose. Frying garlic resulted in a significant reduction in almost all sulfur volatile compounds (Table 1). The levels of allyl methyl disulfide, diallyl disulfide, allyl mercaptan and allyl methyl sulfide were decreased by 75 to 99% by frying (Table 1). Other studies have also shown that frying garlic causes a significant loss of sulfur volatile compounds including diallyl disulfide, allyl methyl sulfide and allyl mercaptan [19,20,21]. Frying breaks down the sulfur-containing compounds in garlic, leading to a decrease in its characteristic malodorous odor-producing volatiles.

However, a few volatiles increase when garlic is fried. 2-vinyl-4H-1,3-dithiin increased in fried garlic (Table 1). Other studies have also shown an increase in 2-vinyl-4H-1,3-dithiin [19,20,21]. Allicin transforms into vinyl dithiins in non-polar solvent so 2-vinyl-4H-1,3-dithiin was expected to increase when the garlic was fried in oil [22]. 2,3-Dimethylpyrazine and pyridine also significantly increased in fried garlic (Table 1). When garlic is fried, nitrogen-containing compounds (pyridine and pyrazine) are formed by decomposing sulfur-containing compounds during heating due to the Maillard reaction [19,23,24].

### 2.2. Effect of Yogurt on Raw and Fried Garlic Volatiles

Because of variability in raw material and the frying process, each treatment was compared to a control made from the same batch of garlic to accurately determine the extent of deodorization by the treatment. The addition of yogurt significantly reduced the concentration of almost all of the volatiles in both raw and fried garlic (Table 2). Yogurt produced a higher percent reduction in raw garlic than fried garlic. In raw garlic, the concentration of diallyl disulfide, allyl mercaptan, allyl methyl sulfide and allyl methyl disulfide were reduced by 99%, while in fried garlic, they were reduced by 82–94%. The volatiles were at a much higher concentration in the raw garlic than in the fried, which may explain why a greater reduction was achieved in the raw garlic.

The yogurt interacts with the sulfur volatiles in garlic, decreasing the concentration of garlic volatiles. We theorize that the reduction was due to the fat, proteins and water present in the yogurt. When garlic is treated with milk, there is a reduction in the concentration of sulfur-containing volatiles [12,13]. The authors proposed that fat and water were responsible for deodorizing the garlic volatiles [12,13,14]. As expected, yogurt shows a similar effect on garlic volatiles to milk. Therefore, this study further focused on each of the components of the yogurt separately to understand the impact of each component on garlic volatiles.

### 2.3. Effect of Fat Versus Protein on Raw and Fried Garlic Volatiles 

**Figure 1:**
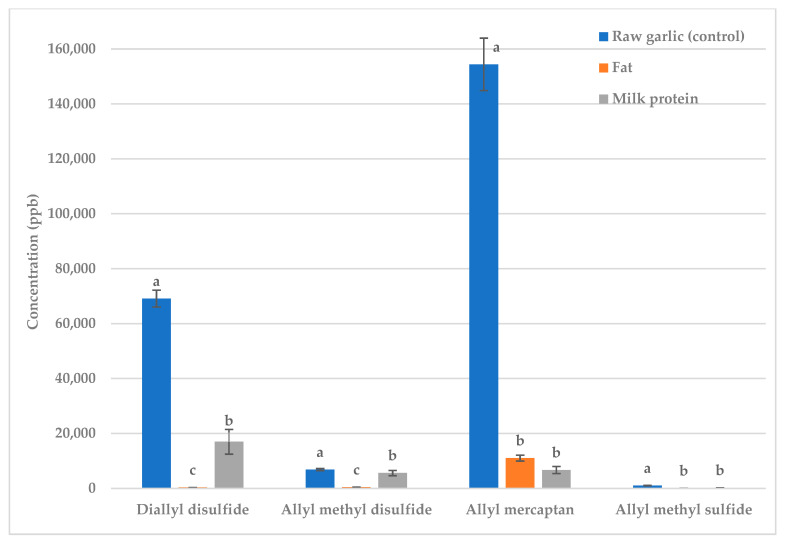
Raw garlic: effect of fat vs. protein on key sulfur volatiles. Treatments within the same volatile with different letters are significantly different (*p* < 0.05).

**Figure 2:**
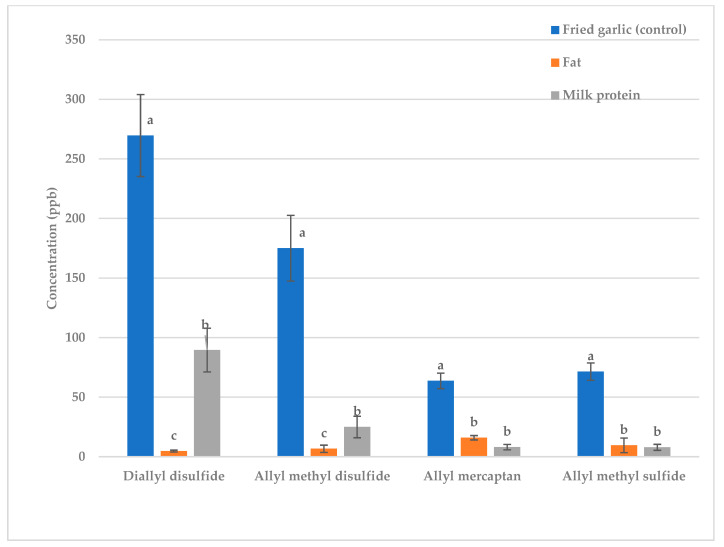
Fried garlic: effect of fat vs. protein on key sulfur volatiles. Treatments within the same volatile with different letters are significantly different (*p* < 0.05).

Next, 9% butter fat or milk protein were added to raw garlic and fried garlic (Figure 1 and Figure 2). The concentrations of almost all sulfur- containing volatiles were greatly reduced; thus, both fat and protein are excellent treatments for reducing the malodorous odor produced by garlic. 

Whether fat or protein was more effective at deodorization depended on the volatile (Figure 1 and Figure 2). For diallyl disulfide and allyl methyl disulfide, butter reduced the concentration more than milk protein in both raw and fried garlic (Figure 1 and Figure 2). Diallyl disulfide (LogP = 2.2) and allyl methyl disulfide (LogP = 1.6) are more hydrophobic volatiles so should have stronger interactions with fat than protein [12]. There was no significant difference for allyl mercaptan (LogP = 0.5) and allyl methyl sulfide (LogP = 1.5), but protein appeared to reduce the concentration more than fat in both raw and fried garlic. Allyl mercaptan and allyl methyl sulfide are less hydrophobic in nature so they should have stronger interactions with the protein than with the fat [12].

### 2.4. Effect of Water and Protein on Raw and Fried Garlic Volatiles

**Figure 3:**
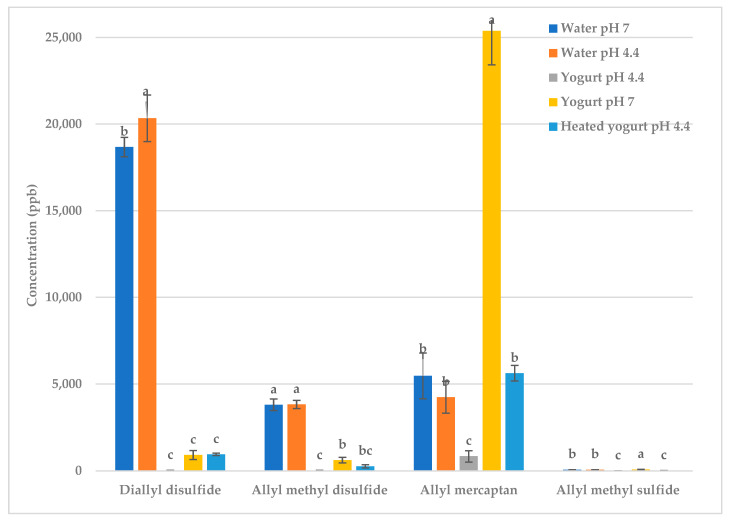
Raw garlic: effect of water, pH and heating on key sulfur volatiles. Treatments within the same volatile with different letters are significantly different (*p* < 0.05).

**Figure 4:**
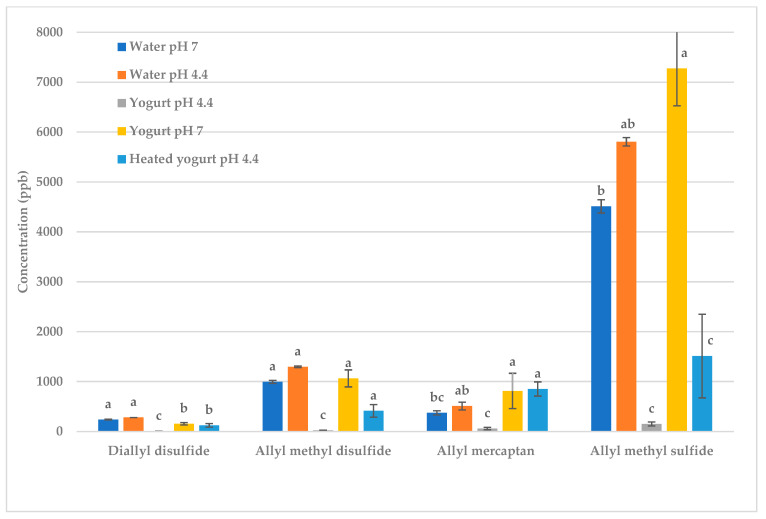
Fried garlic: effect of water, pH and heating on key sulfur volatiles. Treatments within the same volatile with different letters are significantly different (*p* < 0.05).

Water (pH 7) and water adjusted to pH 4.4 were added to garlic to isolate the effect of water and pH (Table A1 and Table A2, Figure 3 and Figure 4). When water was added to the garlic, volatile levels decreased, as expected (Table A1 and Table A2). The reduction in the concentration of volatiles is due to the partitioning of volatiles between phases [12]. The headspace concentrations of these volatiles were lower when water was added to the garlic because the volatiles were partitioning between the garlic, water and gas phases, whereas the volatiles in garlic without water only partitioned between the garlic and gas phases [12,25]. Changing the pH of the water from 7 to 4.4 created no significant difference in the deodorization of the volatiles; thus, the pH, in and of itself, did not affect the volatiles.

Yogurt (pH 4.4) deodorized the garlic, resulting in a high level of reduction (99%) in allyl mercaptan, allyl methyl disulfide, allyl methyl sulfide and diallyl disulfide (Table A1 and Table A2). However, increasing the pH of the yogurt to pH 7 decreased the deodorization (Figure 3 and Figure 4). Figure 3 and Figure 4 show that changing the pH of water between 4.4 and 7 had no effect; thus, this effect was not due to the pH itself. Instead, the pH changed the conformation of the protein, which changed the protein’s ability to bind the volatiles. The functional properties of proteins are highly dependent on their three-dimensional structure, which is affected by pH [13,26,27]. At casein’s isoelectric point, pH 4.6, casein forms a three-dimensional protein network called micellar casein [28]. Micellar casein binds to denatured whey protein via disulfide bridges [28,29]. We propose that the mechanism responsible for deodorization is the casein micelle–whey protein complex binding to the volatiles. The hydrophobic and reactive thiol groups of denatured whey protein (i.e., α-lactalbumin and β-lactoglobulin) are exposed, which allows them to bind with the volatiles [29,30,31,32]. Casein also binds to the volatiles through hydrophobic bonding, hydrogen bonding, covalent interaction, redox reactions or interchange reactions between the thiol and disulfide groups of the sulfur volatiles and the sulfhydryl groups of the protein [13,33,34,35,36]. This casein micelle–whey protein complex binds to more volatiles than either protein separately.

As the pH is increased to 7, the casein micelle–whey protein complex breaks down. At pH 7, casein carries a net negative charge, which results in an electrostatic repulsion between the casein molecules, preventing the formation of the micelle structure. Whey protein is in a stable soluble state, which restricts its binding ability [27,37]. Due to these factors, both proteins were less able to bind the volatiles at pH 7.

The yogurt (pH 4.4) was also heated to 80 °C to denature the proteins. Heating the yogurt resulted in less deodorization of garlic volatiles as compared to unheated yogurt (pH 4.4). When yogurt is heated, the increase in kinetic energy disrupts the bonds in the proteins, resulting in the unfolding and denaturation of both casein and whey protein [13,38]. This decreases the ability of the proteins to bind to volatiles, thus increasing the concentration of volatiles in the headspace.

### 2.5. Effect of Different Proteins on Raw and Fried Garlic Volatiles

**Figure 5:**
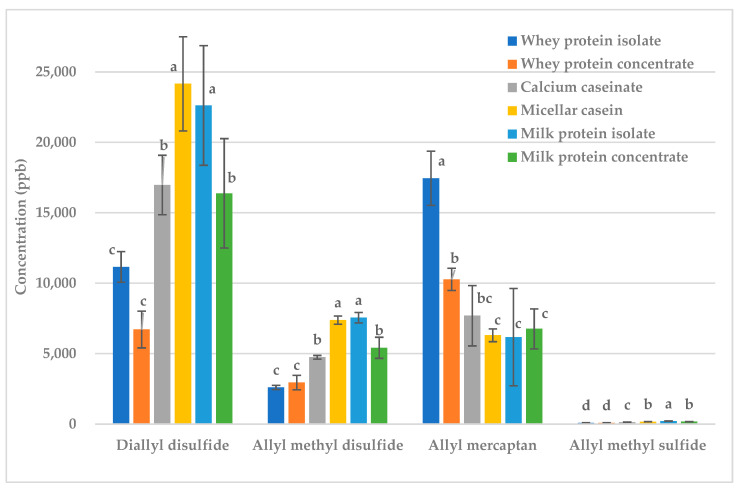
Raw garlic: effect of different proteins on key sulfur volatiles. Treatments within the same volatile with different letters are significantly different (*p* < 0.05).

**Figure 6:**
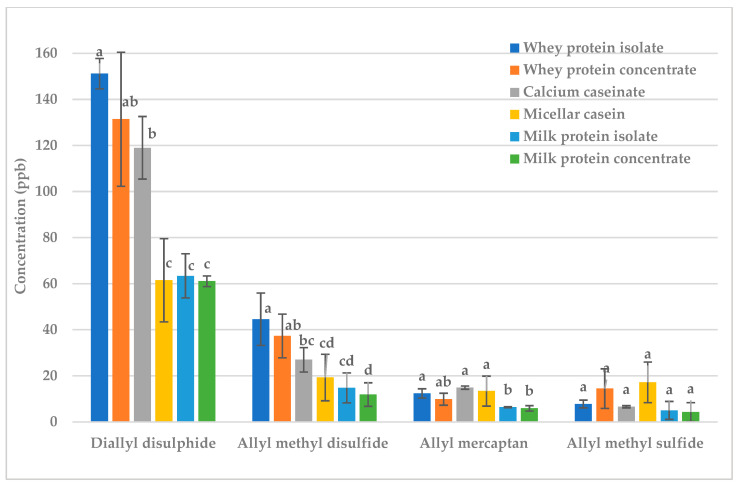
Fried garlic: effect of different proteins on key sulfur volatiles. Treatments within the same volatile with different letters are significantly different (*p* < 0.05).

Two forms of whey protein, casein or milk protein, were mixed with either raw or fried garlic. All of the proteins caused significant deodorization of almost all volatiles (Table A3 and Table A4, Figure 5 and Figure 6). Proteins react with volatiles through a number of mechanisms, including reversible binding, such as hydrogen bonds, hydrophobic interactions and ionic bonds, and irreversible binding via covalent linkages [13].

The type of protein that was most effective at deodorization was different for raw versus fried garlic (Figure 5 and Figure 6). In raw garlic, whey protein created the greatest reduction in volatile concentration, followed by casein then milk protein. In fried garlic, whey protein was less effective than in raw garlic. Frying causes both the Maillard reaction and oxidation of fatty acids in the frying oil, producing many new compounds such as aldehydes, ketones and alcohols. We propose that the whey protein may have preferentially bound to the compounds formed during frying over the sulfur volatiles. The whey protein caused deodorization in both samples, but it was more effective in the raw garlic. 

The form of the protein generally made no significant difference, but when there was a difference, the concentrate was more effective than the isolate. At first, it would logically seem that the isolate, with a higher protein content, would create greater deodorization. However, during the production of concentrates, the proteins undergo less processing than for isolates. Additional processes for the purification and separation of isolates are used. Due to this additional processing, some of the protein structure is changed or denatured, and thus the structures of the proteins are more intact in concentrates than in isolates [39]. The more intact conformation of the proteins in concentrates may have made them more effective in binding the volatiles than the isolates. 

### 2.6. Effect of Quantity of Butter Fat on Raw and Fried Garlic Volatiles 

**Figure 7:**
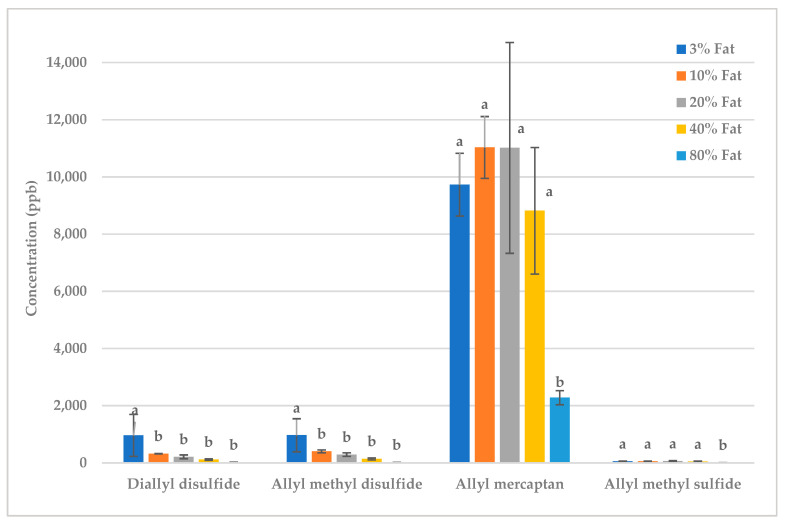
Raw garlic: effect of quantity of fat on key sulfur volatiles. Treatments within the same volatile with different letters are significantly different (*p* < 0.05).

**Figure 8:**
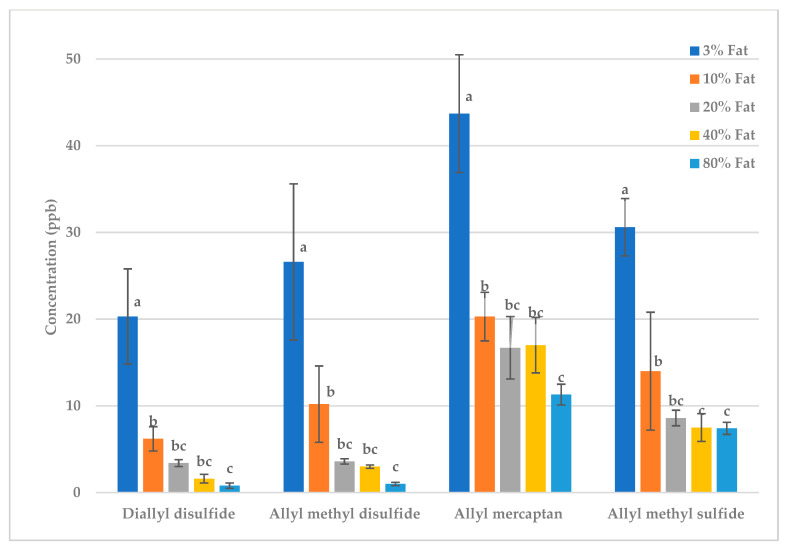
Fried garlic: effect of quantity of fat on key sulfur volatiles. Treatments within the same volatile with different letters are significantly different (*p* < 0.05).

The quantity of butter also affects the deodorization of malodor- producing garlic volatiles. Garlic was mixed with a solution of water containing 3, 10, 20, 40 or 80% butter. Both water and fat reduce volatile levels. The addition of butter to the water significantly increased the deodorization of both raw and fried garlic sulfur volatiles (Table A5 and Table A6, Figure 7 and Figure 8). In both raw and fried garlic, the reduction in the concentration of these four volatiles significantly increased as the butter content increased to 80% (Figure 7 and Figure 8). In raw garlic, many of the treatments were not significantly different. However, in fried garlic, increasing the butter from 3 to 10% created a large decrease, with a significant additional decrease as the butter content further increased. Increasing butter content decreases the concentration of the volatiles by creating a more hydrophobic environment. Sulfur volatiles interact with fat through hydrophobic interactions involving the nonpolar regions of the sulfur volatiles and the fat molecules [9,12,14,40,41]. When butter is added to garlic, these volatiles prefer to reside in the fat phase, thereby lowering their concentration in the headspace [22]. Therefore, using fat is an effective way to minimize the pungent odor of garlic.

## 3. Materials and Methods

### 3.1. Garlic Sample Preparation

Garlic cloves (Walmart, Columbus, OH, USA) were peeled by hand. Using a knife, peeled garlic cloves were cut into slices of approximately 1 mm thickness. Sliced garlic was held for 30 min at room temperature before frying or addition of treatments. Fried garlic was stir-fried in hot canola oil (Kroger, Columbus, OH, USA), (125 °C). A temperature drop was seen immediately after the addition of chopped garlic into the hot oil. The frying continued until the temperature reached 125 °C again, at approximately 12 min. The oil was removed from the fried garlic using tissues and the garlic was cooled at room temperature for 15 min.

### 3.2. Treatment Preparation

Different treatments were used for the determination of the deodorization of the garlic. Because of variability in raw material and the frying process, one batch of garlic was prepared and used for one set of treatments with its control. Thus, there was a different control for each set of treatments (fat and protein, pH of water and yogurt, types of protein, amount of fat). For water, 100 mL water at pH 4.4 or pH 7 and 100 g whole milk plain yogurt (Dannon, Target, Columbus, OH, USA) at pH 4 or pH 7 were used. To adjust the pH, hydrochloric acid was used for acidic pH and sodium hydroxide was used for basic pH. Heating involved briefly immersing the raw or fried garlic mixed with whole fat yogurt (Dannon, Target, Columbus, OH, USA) packed in a Ziplock bag in boiling water, followed by rapid cooling. For protein, a mixture of 50 mL water and 50 g non-fat dry milk powder (Kroger, Columbus, OH, USA) was used. For different proteins, 9% whey protein isolate (Mill Haven Foods, New Lisbon, WI, USA), whey protein concentrate (Mill Haven Foods, New Lisbon, WI, USA), calcium caseinate (Mill Haven Foods, New Lisbon, WI, USA), micellar casein (Mill Haven Foods, New Lisbon, WI, USA), milk protein isolate (Mill Haven Foods, New Lisbon, WI, USA) or milk protein concentrate (Mill Haven Foods, New Lisbon, WI, USA) was used. The proteins were rehydrated by mixing using a magnetic stirrer for 4 h. For the quantity of fat, a 100 mL solution of water containing 3, 10, 20, 40 or 80% of unsalted butter (Kroger, Columbus, OH, USA) was heated, mixed, then cooled to room temperature before using. The raw or fried garlic (6 g) mixed with 100 mL of each treatment was placed in a 500 mL Pyrex bottle. The raw or fried garlic (6 g), was used as a control.

### 3.3. Selected-Ion Flow-Tube Mass Spectrometry (SIFT-MS) Headspace Analysis

The raw or fried garlic (6 g) was mixed with 100 mL of each treatment. The sample was placed in a 500 mL Pyrex bottle capped with a polytetrafluoroethylene (PTFE)- faced silicone septa cap and held for 30 min at room temperature. The headspace volatiles of the garlic sample were measured via an 18-gauge passivated needle connected to the inlet of the selected-ion flow-tube mass spectrometry (SIFT-MS) machine (SYFT Voice200ultra, Syft Ltd., Christchurch, New Zealand) using a selected ion mode (SIM) scan at 20 °C ± 2 and the SIFT-MS headspace method. The SIFT-MS was calibrated with the standard gases benzene, toluene, isobutane, ethylene, tetrafluorobenzene, hexafluorobenzene and octafluorobenzene. Syft VOICE-200 software (v.1.4.9.17754, Syft Technologies Ltd., Christchurch, New Zealand) was used to analyze the volatiles. 2-vinyl-4H-1,3-dithiin is a mixture of 2-vinyl-4H-1,3-dithiin and 3-vinyl-4H-1,2-dithiin, and 2,3-dimethylpyrazine is a mixture of 2,3-dimethylpyrazine, 2,5-dimethylpyrazine and 2,6-dimethylpyrazine. Three replicates were conducted for each deodorizing agent. A blank was used before testing the samples. An empty 500 mL Pyrex bottle was used as the blank. The scanning time for each headspace reading was 95 s. Volatiles tested are given in Table 3.

### 3.4. Statistical Analysis

The data were coded, entered and analyzed using JMP^®^ Pro version 16.0.0 (512340) (Statistical Discovery, Cary, NC, USA). Three replicates of data for each test were obtained. Data were analyzed using one-way analysis of variance (ANOVA) and comparison of all pairs was performed using Fisher’s least significance difference (LSD). Significance was defined as *p* ≤ 0.05.

## 4. Conclusions

Frying garlic significantly reduced the concentration of almost all sulfur volatile compounds. Yogurt also significantly reduced the headspace concentration of all major odor- producing garlic volatiles, including allyl methyl disulfide, diallyl disulfide, allyl mercaptan and allyl methyl sulfide, in both raw and fried garlic. The components responsible for the deodorization of the volatiles were fat, protein and water. At the same concentration, fat was a more effective treatment for the reduction in diallyl disulfide and allyl methyl disulfide, whereas protein seemed to be a more effective treatment for the reduction in allyl methyl sulfide and allyl mercaptan. The proteins whey protein isolate, whey protein concentrate, micellar casein, calcium casein, milk protein isolate and milk protein concentrate were all effective in deodorizing the garlic volatiles. The concentrate form of proteins was more effective than the isolate form in the deodorization of volatiles. pH has no effect on the deodorization of garlic volatiles but affects the proteins by changing the conformation of the protein, which changes the binding of volatiles. Heating yogurt decreases its ability to deodorize the volatiles as heating denatures proteins, which results in weakening of the bonds of the proteins, leading to less deodorization. The amount of fat also affects the concentration of volatiles, with higher quantities of fat making a more hydrophobic environment, leading to more effective deodorization. The results suggest that consuming yogurt or foods with proteins and fat may help reduce the bad odor produced by garlic. Follow- up studies will focus on the effectiveness of these treatments at deodorizing the breath after the consumption of garlic. Protein and fat can be used as a natural deodorizer to mask the strong smell of garlic in processed foods. New products can be formulated to minimize the pungent odor of garlic using fat and dairy proteins.

## Figures and Tables

**Table 1:** Effect of frying on garlic volatiles (concentration (ppb) ± SD).

Volatiles	Raw Garlic	Fried Garlic
1,3-dithiane	1240 ± 879	65.9 ± 31.8
2-ethylpyrazine	7.20 ± 5.83	11.0 ± 4.5
2-methyl-2-butenal	60.9 ± 49.1	23.6 ± 8.67
2-methylbenzaldehyde	5.97 ± 3.37	1.36 ± 0.77
2-vinyl-4H-1,3-dithiin	4.21 ± 2.94	8.26 ± 3.26
2,3-dimethylpyrazine	4.81 ± 3.89	15.5 ± 10.7
2,5-dimethylthiophene	134 ± 100	9.85 ± 1.62
Acetaldehyde	19,100 ± 11,300	7990 ± 3390
Allicin	1330 ± 1190	81.4 ± 42.1
Allyl mercaptan	116,000 ± 20,200	121 ± 8
Allyl methyl disulfide	13,400 ± 8780	79.8 ± 29.2
Allyl methyl sulfide	1380 ± 385	73.3 ± 9.3
Allyl methyl tetrasulfide	2.56 ± 1.25	1.66 ± 0.57
Allyl methyl thiosulfinate	89.4 ± 73.8	8.06 ± 2.02
Allyl methyl trisulfide	35.7 ± 18.5	7.46 ± 0.76
Aniline	2.56 ± 1.56	5.93 ± 3.34
Diallyl disulfide	26,200 ± 19,000	69.1 ± 43.5
Diallyl sulfide	1900 ± 711	51.8 ± 5.7
Diallyl tetrasulfide	2.80 ± 2.12	1.18 ± 0.51
Diallyl trisulfide	236 ± 175	14.7 ± 4.2
Dimethyl disulfide	554 ± 367	47.9 ± 24.8
Dimethyl sulfide	981 ± 692	384 ± 159
Dimethyl thioether	1130 ± 794	447 ± 174
Dimethyl thiosulfinate	10.7 ± 4.7	3.63 ± 0.08
Dimethyl trisulfide	10.9 ± 4.94	9.58 ± 4.06
Dipropyl sulfide	5130 ± 4240	16.3 ± 4.1
Methyl mercaptan	24,400 ± 3580	55.9 ± 7.15
Methyl propyl disulfide	1970 ± 1350	19.5 ± 9.9
Pyridine	2.42 ± 1.65	6.91 ± 3.19
Trimethylpyrazine	493 ± 344	6.14 ± 2.86

**Table 2:** Effect of yogurt on raw and fried garlic (concentration (ppb) ± SD).

Volatiles	Raw Garlic	Yogurt + Raw Garlic	Fried Garlic	Yogurt + Fried Garlic
1,3-dithiane	4020 ± 1770	20.4 ± 6.6	203 ± 2	16.6 ± 15.6
2-ethylpyrazine	29.9 ± 15.7	0.67 ± 0.10	25.4 ± 4.6	1.08 ± 0.76
2-methyl-2-butenal	93.0 ± 37.9	2.36 ± 0.39	36.3 ± 1.5	3.59 ± 2.81
2-methylbenzaldehyde	51.6 ± 25.1	4.20 ± 0.50	2.32 ± 0.09	2.91 ± 2.11
2-vinyl-4H-1,3-dithiin	8.59 ± 3.22	3.18 ± 1.02	13.0 ± 0.2	1.76 ± 0.97
2,3-dimethylpyrazine	38.8 ± 17.1	0.44 ± 0.06	51.9 ± 3.1	0.95 ± 0.84
2,5-dimethylthiophene	401 ± 212	2.59 ± 0.54	19.9 ± 0.7	1.77 ± 1.10
Acetaldehyde	10,400 ± 617	9210 ± 455	3380 ± 322	6770 ± 5310
Allicin	93.9 ± 39.9	3.66 ± 2.73	3.86 ± 1.03	0.41 ± 0.24
Allyl mercaptan	110,000 ± 28,600	832 ± 324	169 ± 5	29.0 ± 27.8
Allyl methyl disulfide	20,100 ± 6150	23.2 ± 8.1	350 ± 18	18.1 ± 16.2
Allyl methyl sulfide	965 ± 275	8.39 ± 0.34	742 ± 20	75.3 ± 90.1
Allyl methyl tetrasulfide	9.34 ± 4.43	2.12 ± 0.30	2.90 ± 0.16	1.27 ± 0.17
Allyl methyl thiosulfinate	50.8 ± 23.8	0.98 ± 0.69	28.9 ± 1.9	1.29 ± 0.83
Allyl methyl trisulfide	35.3 ± 14.6	1.13 ± 0.15	54.0 ± 2.4	3.54 ± 2.78
Aniline	9.38 ± 3.67	0.49 ± 0.09	15.7 ± 2.6	0.29 ± 0.27
Diallyl disulfide	102,000 ± 56,900	28.3 ± 8.7	84.9 ± 2.3	4.64 ± 3.92
Diallyl sulfide	1720 ± 841	5.96 ± 2.40	250 ± 14	18.1 ± 21.8
Diallyl tetrasulfide	13.3 ± 6.03	3.38 ± 0.11	1.44 ± 0.23	1.20 ± 0.66
Diallyl trisulfide	981 ± 522	2.35 ± 0.79	28.4 ± 1.8	2.11 ± 1.49
Dimethyl disulfide	487 ± 71.3	2.93 ± 0.92	293 ± 3	23.1 ± 27.6
Dimethyl sulfide	2870 ± 1350	69.7 ± 4.21	151 ± 16	37.0 ± 33.8
Dimethyl thioether	2990 ± 950	77.6 ± 7.43	182 ± 43	43.4 ± 40.3
Dimethyl thiosulfinate	13.5 ± 6.8	1.00 ± 0.51	15.8 ± 0.2	0.81 ± 0.26
Dimethyl trisulfide	10.6 ± 1.6	2.24 ± 0.53	110 ± 15	7.00 ± 5.40
Dipropyl sulfide	7900 ± 2530	30.5 ± 4.40	194 ± 15	23.6 ± 19.2
Methyl mercaptan	16,600 ± 1300	449 ± 172	218 ± 29	102 ± 103
Methyl propyl disulfide	3410 ± 1130	4.92 ± 0.83	71.1 ± 1.7	4.07 ± 3.59
Pyridine	20.8 ± 6.6	0.78 ± 0.11	16.6 ± 0.8	0.76 ± 0.65
Trimethylpyrazine	958 ± 364	1.80 ± 0.33	24.0 ± 1.5	1.38 ± 0.96

**Table 3:** Properties of volatiles tested in selected-ion flow-tube mass spectrometry (SIFT-MS) headspace analysis.

Volatile Compound	Ion Product	Reagent Ion	*m*/*z*	Reaction Rate (k) 10^−9^ cm^3^ s^−1^
1-3-dithiane	C_4_H_7_S^+^	O_2_^+^	87	2.3
2-ethylpyrazine	C_6_H_8_N_2_.H^+^	H_3_O^+^	109	3
2-Methyl-2-butenal	C_5_H_7_O^+^	NO^+^	83	4
2-methylbenzyaldehyde	C_8_H_7_O^+^	NO^+^	119	3.3
2-vinyl-4H-1,3-dithiin	C_6_H_8_S_2_^+^	NO^+^	144	2.4
2,3-dimethylpyrazine	C_6_N_2_H_8_.H^+^	H_3_O^+^	109	3.4
2,5-dimethylthiophene	C_6_H_8_S.H^+^	H_3_O^+^	113	3
Acetaldehyde	CH_3_CO^+^	NO^+^	43	6
	C_2_H_5_O^+^	H_3_O^+^	45	3.7
Allicin	C_6_H_10_OS_2_	NO^+^	162	2.4
Allyl mercaptan	C_3_H_6_S	NO^+^	74	2.4
	C_3_H_6_S.H^+^	H_3_O^+^	75	2.6
Allyl methyl disulfide	C_4_H_8_S_2_	NO^+^	120	2.4
	C_4_H_8_S_2_.H^+^	H_3_O^+^	121	2.6
Allyl methyl sulfide	C_4_H_8_S^+^	NO^+^	88	2.5
	C_4_H_8_S.H^+^	H_3_O^+^	89	3
Allyl methyl tetrasulfide	C_4_H_8_S_4_^+^	NO^+^	184	2.4
Allyl methyl thiosulfinate	C_4_H_8_S_2_O	NO^+^	136	2.4
Allyl methyl trisulfide	C_4_H_8_S_3_^+^	NO^+^	152	2.4
Aniline	C_6_H_5_NH_2_.H^+^	H_3_O^+^	94	2.8
Diallyl disulfide	(C_3_H_5_)_2_S_2_.H^+^	H_3_O^+^	147	3
Diallyl sulfide	(C_3_H_5_)_2_S.H^+^	H_3_O^+^	115	2.9
Diallyl tetrasulfide	C_6_H_10_S_4_^+^	NO^+^	210	2.4
Diallyl trisulfide	C_6_H_10_S_3_	NO^+^	178	2.4
Dimethyl disulfide	(CH_3_)_2_S_2_.H_+_	H_3_O^+^	95	2.6
Dimethyl sulfide	(CH_3_)_2_S^+^	O_2_^+^	62	2.2
Dimethyl thioether	(CH_3_)_2_S^+^	O_2_^+^	62	2.2
	(CH_3_)_2_S^+^	NO^+^	62	2.2
Dimethyl thiosulfinate	C_2_H_6_OS_2_^+^	NO^+^	110	2.4
Dimethyl trisulfide	C_2_H_6_S_3_^+^	NO^+^	126	1.9
Dipropyl sulfide	C_3_H_4_S^+^	O_2_^+^	72	2.4
Methyl mercaptan	CH_4_S.H^+^	H_3_O^+^	49	1.8
Methyl propyl disulfide	C_4_H_10_S_2_^+^	NO^+^	122	1.5
	C_4_H_10_S_2_.H^+^	H_3_O^+^	123	2.5
Pyridine	C_5_H_5_N.H^+^	H_3_O^+^	80	3.3
Trimethylpyrazine	C_7_H_10_N_2_^+^	O_2_^+^	122	2.5

## Data Availability

The data presented in this study are available in Appendix A.

## Appendix A

**Table A1:** Raw garlic: effect of pH and heating on the volatiles.

Volatiles	Garlic (Control)	Yogurt pH 4.4	Yogurt pH 7	Water pH 4.4	Water pH 7	Heated Yogurt pH 4.4
1,3-dithiane	4020 ± 1770	20.4 ± 6.6	154 ± 24	589 ± 30	524 ± 8	71.5 ± 8.5
2-ethylpyrazine	29.9 ± 15.7	0.67 ± 0.10	1.49 ± 0.12	3.35 ± 0.13	3.81 ± 1.32	1.65 ± 0.15
2-methyl-2-butenal	93 ± 37	2.36 ± 0.39	4.44 ± 0.48	7.85 ± 0.35	6.05 ± 0.91	3.04 ± 0.46
2-methylbenzaldehyde	51.6 ± 25.1	4.2 ± 0.5	5.85 ± 1.10	2.29 ± 0.49	1.89 ± 0.23	5.62 ± 0.67
2-vinyl-4H-1,3-dithiin	8.59 ± 3.22	3.18 ± 1.02	4.66 ± 1.02	2.38 ± 0.75	3.61 ± 0.87	5.42 ± 0.74
2,3-dimethylpyrazine	38.8 ± 17.1	0.44 ± 0.06	3.91 ± 0.90	2.24 ± 0.09	2.55 ± 0.88	1.89 ± 0.34
2,5-dimethylthiophene	401 ± 212	2.59 ± 0.54	14.6 ± 1.4	85.4 ± 9.84	78.4 ± 2.48	15.7 ± 3.5
Acetaldehyde	10,400 ± 617	9210 ± 455	7390 ± 273	1720 ± 237	1190 ± 617	6722 ± 419
Allicin	93.9 ± 39.9	3.66 ± 2.73	2.97 ± 0.61	35.8 ± 3.3	29.4 ± 1.6	9.58 ± 5.20
Allyl mercaptan	110,000 ± 28,600	832 ± 324	25,400 ± 1950	4240 ± 915	5470 ± 1320	5621 ± 450
Allyl methyl disulfide	20,100 ± 6150	23.2 ± 8.1	615 ± 159	3820 ± 236	3800 ± 335	262 ± 97
Allyl methyl sulfide	965 ± 275	8.39 ± 0.34	79.9 ± 11.8	63.2 ± 4.8	63.8 ± 5.5	17.4 ± 3.8
Allyl methyl tetrasulfide	9.34 ± 4.43	2.12 ± 0.30	1.7 ± 1.1	3.13 ± 0.51	2.77 ± 0.87	2.08 ± 0.18
Allyl methyl thiosulfinate	50.8 ± 23.8	0.98 ± 0.69	1.06 ± 0.26	5.81 ± 1.54	4.86 ± 0.88	1.71 ± 0.55
Allyl methyl trisulfide	35.3 ± 14.6	1.13 ± 0.15	2.00 ± 0.11	15.6 ± 0.4	13.5 ± 2.6	1.52 ± 0.20
Aniline	9.38 ± 3.67	0.49 ± 0.09	1.04 ± 0.03	1.10 ± 0.26	1.51 ± 0.22	0.57 ± 0.05
Diallyl disulfide	102,000 ± 56,900	28.3 ± 8.7	907 ± 251	20,300 ± 1350	18,700 ± 556	945 ± 73
Diallyl sulphide	1720 ± 841	5.96 ± 2.40	76.7 ± 14.4	243 ± 27	189 ± 4	46.9 ± 6.4
Diallyl tetrasulfide	13.3 ± 6.0	3.38 ± 0.11	3.63 ± 0.62	5.74 ± 1.76	6.29 ± 2.00	3.65 ± 0.61
Diallyl trisulfide	981 ± 522	2.35 ± 0.79	9.55 ± 3.60	350 ± 18	323 ± 21	11.0 ± 1.5
Dimethyl disulphide	487 ± 71	2.93 ± 0.92	50.4 ± 9.7	99.0 ± 10.6	98.7 ± 12.6	9.20 ± 2.97
Dimethyl sulfide	2870 ± 1350	69.7 ± 4.2	109 ± 16	815 ± 49	560 ± 271	72.1 ± 5.4
Dimethyl thioether	2990 ± 950	77.6 ± 7.4	109 ± 16	846 ± 122	567 ± 335	72.1 ± 5.4
Dimethyl thiosulfinate	13.5 ± 6.8	1.00 ± 0.51	2.10 ± 0.93	1.16 ± 0.41	1.24 ± 0.23	0.87 ± 0.13
Dimethyl trisulfide	10.6 ± 1.6	2.24 ± 0.53	2.56 ± 1.23	3.11 ± 0.43	3.20 ± 1.82	2.30 ± 0.21
Dipropyl sulfide	7900 ± 2530	30.5 ± 4.4	440 ± 58	2220 ± 139	2060 ± 50	179.0 ± 13.0
Methyl mercaptan	16,600 ± 1300	449 ± 172	6280 ± 394	1590 ± 262	1900 ± 243	1061 ± 2
Methyl propyl disulfide	3410 ± 1130	4.92 ± 0.83	80.9 ± 22.1	588 ± 37	519 ± 28	37.6 ± 9.4
Pyridine	20.8 ± 6.6	0.78 ± 0.11	2.30 ± 0.64	2.13 ± 0.20	3.13 ± 0.41	1.22 ± 0.27
Trimethylpyrazine	958 ± 364	1.80 ± 0.33	23.7 ± 5.6	160 ± 18	147 ± 15	10.8 ± 3.08

**Table A2:** Fried garlic: effect of pH and heating on the volatiles.

Volatiles	Fried Garlic (Control)	Yogurt pH 4.4	Yogurt pH 7	Water pH 4.4	Water pH 7	Heated Yogurt pH 4.4
1,3-dithiane	119 ± 29	11.5 ± 5.6	572 ± 148	543 ± 60	430 ± 32	310 ± 103
2-ethylpyrazine	31.2 ± 7.3	0.91 ± 0.42	27.4 ± 9.1	12.9 ± 1.8	10.0 ± 1.8	4.66 ± 1.96
2-methyl-2-butenal	39.8 ± 10.4	4.66 ± 1.53	61.8 ± 15.6	34.0 ± 6.4	27.7 ± 3.8	13.6 ± 7.32
2-methylbenzaldehyde	3.21 ± 0.78	3.77 ± 0.72	5.98 ± 1.14	2.89 ± 0.53	2.15 ± 0.35	5.97 ± 1.15
2-vinyl-4H-1,3-dithiin	19.9 ± 4.1	2.23 ± 0.22	18.4 ± 8.2	11.5 ± 0.6	10.3 ± 0.9	5.55 ± 1.39
2,3-dimethylpyrazine	32.2 ± 9.1	0.71 ± 0.13	20.1 ± 6.9	9.07 ± 1.01	7.06 ± 1.24	3.48 ± 1.93
2,5-dimethylthiophene	19.1 ± 5.2	2.56 ± 0.82	35.8 ± 8.4	48.0 ± 4.2	39.1 ± 1.4	15.1 ± 5.6
Acetaldehyde	7490 ± 356	11,500 ± 1290	7300 ± 1780	1330 ± 113	1140 ± 68	10,700 ± 917
Allicin	6.65 ± 1.75	0.93 ± 0.16	10.5 ± 4.3	9.07 ± 1.13	7.43 ± 0.58	2.37 ± 0.85
Allyl mercaptan	654 ± 56	93.4 ± 44.5	1290 ± 647	809 ± 153	595 ± 76	350 ± 141
Allyl methyl disulfide	142 ± 34	13.1 ± 6.8	615 ± 222	749 ± 102	575 ± 39	586 ± 126
Allyl methyl sulfide	383 ± 60	103 ± 60	4950 ± 1120	3950 ± 663	3070 ± 197	1990 ± 838
Allyl methyl tetrasulfide	2.43 ± 0.97	1.14 ± 0.44	3.86 ± 0.75	5.02 ± 1.83	4.08 ± 1.16	1.89 ± 0.73
Allyl methyl thiosulfinate	19.1 ± 4.3	1.23 ± 0.28	20.1 ± 5.5	14.9 ± 0.7	11.3 ± 1.7	8.50 ± 3.67
Allyl methyl trisulfide	30.1 ± 7.1	1.56 ± 0.08	36.9 ± 9.4	66.5 ± 2.3	57.0 ± 2.0	50.1 ± 12.3
Aniline	17.0 ± 4.4	0.72 ± 0.31	22.0 ± 7.9	8.24 ± 1.22	5.97 ± 0.60	1.97 ± 0.84
Diallyl disulfide	95.1 ± 26.3	4.96 ± 2.36	164 ± 42	297 ± 28	252 ± 14	114 ± 36
Diallyl sulphide	187 ± 40	31.0 ± 18.5	1500 ± 356	1970 ± 127	1620 ± 140	610 ± 211
Diallyl tetrasulfide	2.48 ± 0.68	1.35 ± 0.43	2.48 ± 0.61	3.25 ± 0.72	2.82 ± 0.49	1.84 ± 0.16
Diallyl trisulfide	47.7 ± 12.6	3.21 ± 0.14	36.0 ± 8.55	102 ± 32	83.1 ± 2.7	26.9 ± 9.3
Dimethyl disulphide	91.0 ± 20.3	13.0 ± 7.2	690 ± 179	560 ± 153	407 ± 26	550 ± 219
Dimethyl sulfide	524 ± 57	190 ± 78	1970 ± 243	1280 ± 230	1040 ± 61	259 ± 122
Dimethyl thioether	655 ± 76	223 ± 95	2320 ± 296	1630 ± 359	1360 ± 88	300 ± 140
Dimethyl thiosulfinate	21.4 ± 4.5	1.06 ± 0.32	16.4 ± 6.6	6.95 ± 2.32	5.23 ± 0.11	1.86 ± 0.82
Dimethyl trisulfide	25.6 ± 5.7	4.06 ± 1.20	73.1 ± 17.1	94.2 ± 18.9	69.7 ± 5.9	121 ± 38
Dipropyl sulfide	209 ± 40	34.1 ± 7.5	1180 ± 292	1470 ± 101	1180 ± 101	406 ± 150
Methyl mercaptan	539 ± 54	207 ± 87	2240 ± 1170	1080 ± 420	722 ± 119	523 ± 161
Methyl propyl disulfide	41.6 ± 10.3	3.41 ± 1.34	106 ± 32	119 ± 17	92.2 ± 6.9	83.8 ± 26.5
Pyridine	12.9 ± 2.91	1.38 ± 0.19	11.0 ± 3.9	4.42 ± 0.77	3.46 ± 0.36	2.05 ± 0.60
Trimethylpyrazine	14.2 ± 4.77	1.51 ± 0.42	35.3 ± 9.66	40.3 ± 5.27	30.9 ± 2.2	24.6 ± 9.54

**Table A3:** Raw garlic: effect of different proteins on the volatiles.

Volatiles	Raw Garlic (Control)	Whey Protein Isolate	Whey Protein Concentrate	Calcium Caseinate	Micellar Casein	Milk Protein Isolate	Milk Protein Concentrate
1,3-dithiane	5230 ± 441	371 ± 40	297 ± 48	560 ± 42	824 ± 70	808 ± 113	617 ± 118
2-ethylpyrazine	20.4 ± 2.1	5.67 ± 1.07	4.48 ± 1.04	5.18 ± 1.72	4.10 ± 0.93	4.88 ± 1.44	5.23 ± 1.69
2-methyl-2-butenal	73 ± 4	6.80 ± 0.95	7.86 ± 1.62	8.78 ± 1.62	17.1 ± 5.6	16.9 ± 0.3	11.9 ± 4.4
2-methylbenzaldehyde	70.2 ± 6.7	3.29 ± 0.34	2.55 ± 0.72	3.04 ± 0.99	4.07 ± 0.78	3.84 ± 1.28	3.36 ± 0.48
2-vinyl-4H-1,3-dithiin	8.06 ± 2.07	2.36 ± 0.85	3.74 ± 0.66	3.65 ± 0.66	2.79 ± 0.94	2.82 ± 0.97	3.02 ± 0.57
2,3-dimethylpyrazine	25.6 ± 1.3	8.05 ± 1.26	4.24 ± 0.49	3.46 ± 1.15	2.74 ± 0.62	3.26 ± 0.96	3.49 ± 1.13
2,5-dimethylthiophene	227 ± 18	48.7 ± 5.4	32.8 ± 5.5	70.9 ± 7.8	99.9 ± 10.3	99.3 ± 17.2	74.5 ± 20.7
Acetaldehyde	11,000 ± 1050	2630 ± 442	4690 ± 260	2370 ± 349	3670 ± 1060	3610 ± 367	3320 ± 624
Allicin	28.7 ± 1.4	15.2 ± 1.3	24.8 ± 7.5	19.3 ± 3.3	21.3 ± 4.8	31.0 ± 10.4	30.1 ± 10.6
Allyl mercaptan	154,000 ± 9560	17,500 ± 1920	10,300 ± 794	7690 ± 2140	6310 ± 456	6170 ± 3460	6680 ± 1290
Allyl methyl disulfide	6830 ± 378	2600 ± 150	2950 ± 516	4740 ± 130	7380 ± 295	7550 ± 369	5580 ± 966
Allyl methyl sulfide	1040 ± 86	87.1 ± 8.5	80.3 ± 13.1	135 ± 7	167 ± 3	207 ± 9	173 ± 14
Allyl methyl tetrasulfide	6.08 ± 2.10	1.96 ± 1.47	1.48 ± 0.77	2.46 ± 0.20	2.40 ± 1.32	3.39 ± 0.33	2.72 ± 0.69
Allyl methyl thiosulfinate	55.3 ± 3.9	4.20 ± 1.08	3.68 ± 0.27	4.97 ± 1.03	5.13 ± 0.76	5.03 ± 0.50	4.92 ± 0.18
Allyl methyl trisulfide	21.5 ± 1.6	6.47 ± 1.83	8.27 ± 1.10	13.5 ± 3.6	22.9 ± 1.6	20.5 ± 3.4	18.2 ± 4.36
Aniline	21.8 ± 0.4	1.40 ± 0.20	1.46 ± 0.14	1.92 ± 0.64	3.01 ± 0.91	2.35 ± 0.45	2.19 ± 0.22
Diallyl disulfide	69,100 ± 3100	11,200 ± 1090	6710 ± 1300	17,000 ± 2100	24,200 ± 3340	22,600 ± 4250	17,000 ± 4510
Diallyl sulphide	3080 ± 253	188 ± 13	126 ± 17	386 ± 31	423 ± 35	509 ± 62	381 ± 76
Diallyl tetrasulfide	10.5 ± 1.6	3.82 ± 1.88	4.05 ± 1.09	4.67 ± 1.91	7.78 ± 0.30	5.89 ± 2.68	5.98 ± 0.90
Diallyl trisulfide	566 ± 57	187 ± 14.5	102 ± 26	277 ± 48	389 ± 63	382 ± 105	302 ± 86
Dimethyl disulphide	174 ± 5	73 ± 3.23	141 ± 15	159 ± 16	263 ± 11	309 ± 13	216 ± 23
Dimethyl sulfide	1980 ± 68	468 ± 46.2	309 ± 55	707 ± 66	984 ± 116	938 ± 135	718 ± 161
Dimethyl thioether	2070 ± 123	575 ± 58.8	377 ± 70	913 ± 89	1270 ± 153	1210 ± 179	924 ± 211
Dimethyl thiosulfinate	12.0 ± 1.1	1.16 ± 0.48	0.50 ± 0.18	1.19 ± 0.29	1.02 ± 0.20	0.99 ± 0.36	0.76 ± 0.18
Dimethyl trisulfide	5.53 ± 0.65	2.71 ± 0.36	3.78 ± 0.73	3.83 ± 1.33	4.75 ± 0.44	4.83 ± 0.95	5.89 ± 1.10
Dipropyl sulfide	4480 ± 303	1560 ± 165	884 ± 178	1850 ± 200	2540 ± 322	2610 ± 414	1960 ± 439
Methyl mercaptan	15,300 ± 1330	4370 ± 277	3980 ± 170	3150 ± 401	3080 ± 84	2850 ± 1130	2970 ± 409
Methyl propyl disulfide	1130 ± 51	331 ± 10	373 ± 57	637 ± 21	1020 ± 43	1020 ± 110	735 ± 158
Pyridine	35.8 ± 3.8	3.64 ± 0.57	2.65 ± 0.38	2.88 ± 0.52	2.49 ± 0.54	3.29 ± 0.14	2.70 ± 0.80
Trimethylpyrazine	343 ± 11	96.6 ± 7.52	109 ± 23	171 ± 3	268 ± 10	283 ± 15	202 ± 25

**Table A4:** Fried garlic: effect of different proteins on the volatiles.

Volatiles	Fried Garlic (Control)	Whey Protein Isolate	Whey Protein Concentrate	Calcium Caseinate	Micellar Casein	Milk Protein Isolate	Milk Protein Concentrate
1,3-dithiane	68.2 ± 12.6	37.7 ± 5.9	36.5 ± 8.8	24.8 ± 2.88	17 ± 9.85	12.5 ± 4.7	10.4 ± 4.2
2-ethylpyrazine	2.60 ± 0.62	0.89 ± 0.11	0.93 ± 0.14	0.79 ± 0.28	0.74 ± 0.17	0.50 ± 0.34	0.38 ± 0.13
2-methyl-2-butenal	38.2 ± 8.35	1.92 ± 0.58	1.95 ± 0.64	1.86 ± 0.52	1.66 ± 0.64	1.94 ± 0.81	1.06 ± 0.45
2-methylbenzaldehyde	1.37 ± 0.46	0.52 ± 0.11	0.80 ± 0.11	0.72 ± 0.25	0.58 ± 0.11	0.43 ± 0.28	0.59 ± 0.23
2-vinyl-4H-1,3-dithiin	15.9 ± 1.10	7.94 ± 1.84	5.51 ± 0.27	6.6 ± 1.05	3.62 ± 0.77	4.21 ± 0.31	3.65 ± 0.30
2,3-dimethylpyrazine	1.72 ± 0.40	0.74 ± 0.07	0.61 ± 0.09	0.51 ± 0.18	0.49 ± 0.12	0.34 ± 0.24	0.38 ± 0.25
2,5-dimethylthiophene	17.0 ± 3.45	9.32 ± 0.50	9.22 ± 1.81	7.72 ± 0.5	4.56 ± 0.46	3.86 ± 0.61	3.94 ± 1.10
Acetaldehyde	3340 ± 748	110 ± 5	87.2 ± 2.7	121 ± 15.1	39.3 ± 11.4	50.3 ± 7.5	37.9 ± 7.4
Allicin	5.62 ± 2.79	1.53 ± 0.16	1.61 ± 1.02	1.74 ± 0.47	0.89 ± 0.3	1.48 ± 0.22	0.94 ± 0.38
Allyl mercaptan	46.6 ± 3.30	12.4 ± 2.0	9.88 ± 2.58	14.9 ± 0.7	13.4 ± 6.5	6.35 ± 0.22	5.90 ± 1.17
Allyl methyl disulfide	83.6 ± 15.4	44.6 ± 11.4	37.3 ± 9.4	27 ± 5	19.3 ± 10.1	14.8 ± 6.4	11.9 ± 5.1
Allyl methyl sulfide	38.7 ± 6.53	7.81 ± 1.75	14.5 ± 8.6	6.57 ± 0.53	17.2 ± 18.1	4.99 ± 3.93	4.29 ± 2.26
Allyl methyl tetrasulfide	2.40 ± 1.12	1.79 ± 0.27	1.97 ± 1.06	1.81 ± 0.50	1.34 ± 0.40	1.27 ± 0.12	1.45 ± 0.40
Allyl methyl thiosulfinate	7.72 ± 3.24	2.53 ± 0.71	1.88 ± 0.35	1.97 ± 0.43	1.03 ± 0.44	1.17 ± 0.19	1.03 ± 0.22
Allyl methyl trisulfide	21.2 ± 5.05	19.3 ± 4.1	18.0 ± 2.3	20.4 ± 1.8	8.13 ± 1.29	8.68 ± 1.33	8.90 ± 2.70
Aniline	1.78 ± 0.13	0.33 ± 0.08	0.28 ± 0.10	0.44 ± 0.18	0.36 ± 0.26	0.25 ± 0.17	0.33 ± 0.07
Diallyl disulfide	184 ± 7	151 ± 6.5	131 ± 29	119 ± 13	61.5 ± 8.8	63.4 ± 9.6	61.1 ± 4.1
Diallyl sulphide	48.1 ± 7.94	25.5 ± 3.4	29.1 ± 7.6	15.6 ± 1.35	13.5 ± 7.9	9.87 ± 6.34	5.15 ± 1.02
Diallyl tetrasulfide	2.80 ± 0.55	2.23 ± 0.16	2.06 ± 0.53	3.01 ± 1.00	1.88 ± 0.82	1.77 ± 1.15	2.20 ± 0.60
Diallyl trisulfide	56.5 ± 24.7	46.5 ± 7.2	48.5 ± 10.5	62 ± 3	28.7 ± 3.7	38.2 ± 10.3	32.1 ± 4.9
Dimethyl disulphide	14.7 ± 3.8	22.5 ± 1.4	5.79 ± 2.58	3.38 ± 0.90	5.30 ± 5.82	1.87 ± 0.66	2.45 ± 1.89
Dimethyl sulfide	273 ± 82	21.1 ± 1.3	25.4 ± 4.3	22.1 ± 10.7	10.9 ± 4.3	9.78 ± 1.44	8.75 ± 4.17
Dimethyl thioether	338 ± 107	22.4 ± 3.2	25.4 ± 4.3	25.1 ± 15.8	11.1 ± 4.0	10.3 ± 0.6	8.75 ± 4.17
Dimethyl thiosulfinate	2.62 ± 0.81	0.60 ± 0.26	0.67 ± 0.11	0.74 ± 0.17	0.97 ± 0.43	0.83 ± 0.14	0.71 ± 0.28
Dimethyl trisulfide	12.4 ± 0.5	8.09 ± 3.51	7.94 ± 1.83	6.73 ± 0.31	4.94 ± 3.08	3.75 ± 1.33	3.89 ± 2.03
Dipropyl sulfide	110 ± 6	69.6 ± 3.9	74.1 ± 6.36	66.3 ± 6.99	38.1 ± 6.0	44.5 ± 5.3	37.8 ± 5.06
Methyl mercaptan	17.7 ± 4.4	6.61 ± 0.81	1.70 ± 0.69	12.9 ± 1.04	15.4 ± 7.7	2.73 ± 0.63	2.79 ± 0.01
Methyl propyl disulfide	11.7 ± 2.2	6.19 ± 1.52	7.09 ± 1.75	5.51 ± 0.60	3.36 ± 2.11	2.75 ± 1.02	2.06 ± 0.56
Pyridine	1.66 ± 0.10	0.63 ± 0.22	0.29 ± 0.10	0.54 ± 0.13	0.32 ± 0.17	0.46 ± 0.02	0.45 ± 0.10
Trimethylpyrazine	3.60 ± 0.51	2.32 ± 0.38	1.90 ± 0.14	1.60 ± 0.13	1.20 ± 0.67	0.96 ± 0.33	0.71 ± 0.06

**Table A5:** Raw garlic: effect of quantity of fat on the volatiles.

Volatiles	Raw Garlic (Control)	3% Fat	10% Fat	20% Fat	40% Fat	80% Fat
1,3-dithiane	5230 ± 441	101 ± 40	83.7 ± 17.5	102 ± 37	107 ± 15	11.8 ± 3.4
2-ethylpyrazine	20.4 ± 2.1	2.10 ± 0.57	2.10 ± 0.23	2.41 ± 0.45	1.87 ± 0.23	1.01 ± 0.12
2-methyl-2-butenal	73 ± 4	2.11 ± 0.20	3.26 ± 0.45	4.17 ± 0.33	3.52 ± 0.58	2.91 ± 0.37
2-methylbenzaldehyde	70.2 ± 6.7	1.38 ± 0.33	1.91 ± 0.79	2.52 ± 0.99	2.31 ± 0.50	1.36 ± 0.13
2-vinyl-4H-1,3-dithiin	8.06 ± 2.07	8.74 ± 0.59	8.43 ± 1.13	8.27 ± 2.44	5.12 ± 0.75	3.46 ± 0.56
2,3-dimethylpyrazine	25.6 ± 1.3	3.36 ± 1.00	2.34 ± 0.24	2.73 ± 0.60	2.07 ± 0.97	0.73 ± 0.01
2,5-dimethylthiophene	227 ± 18	25.2 ± 1.8	12.4 ± 0.6	9.03 ± 3.50	5.29 ± 0.70	1.96 ± 0.16
Acetaldehyde	11,000 ± 1050	1640 ± 270	2360 ± 307	3520 ± 544	3680 ± 716	3500 ± 544
Allicin	28.7 ± 1.4	26.1 ± 5.1	24.1 ± 1.2	20.1 ± 2.9	12.5 ± 3.1	6.40 ± 1.34
Allyl mercaptan	154,000 ± 9560	9730 ± 1100	11,000 ± 1080	11,000 ± 3690	8820 ± 2210	2280 ± 247
Allyl methyl disulfide	6830 ± 378	968 ± 579	404 ± 49	291 ± 63	137 ± 32	14.9 ± 2.8
Allyl methyl sulfide	1040 ± 86	55.1 ± 10.8	53.7 ± 9.7	60.8 ± 23.1	45.5 ± 15.2	10.5 ± 0.2
Allyl methyl tetrasulfide	6.08 ± 2.10	1.24 ± 0.49	1.33 ± 0.81	1.28 ± 0.32	0.87 ± 0.35	0.92 ± 0.53
Allyl methyl thiosulfinate	55.3 ± 3.9	20.9 ± 1.4	12.9 ± 0.9	15.0 ± 2.33	14.5 ± 2.5	8.52 ± 0.75
Allyl methyl trisulfide	21.5 ± 1.6	1.70 ± 0.22	1.25 ± 0.76	1.16 ± 0.18	1.14 ± 0.57	1.00 ± 0.46
Aniline	21.8 ± 0.4	0.95 ± 0.17	1.21 ± 0.20	2.69 ± 1.49	2.17 ± 0.76	1.97 ± 0.62
Diallyl disulfide	69,100 ± 3100	964 ± 735	322 ± 5	213 ± 65	119 ± 24	19.5 ± 2.3
Diallyl sulphide	3080 ± 253	45.2 ± 23.6	24.0 ± 3.2	27.3 ± 7.7	38.4 ± 7.6	7.33 ± 1.58
Diallyl tetrasulfide	10.5 ± 1.6	2.92 ± 1.11	3.74 ± 0.14	3.05 ± 0.38	4.06 ± 2.29	1.58 ± 0.41
Diallyl trisulfide	566 ± 57	12.8 ± 8.1	6.13 ± 0.97	3.41 ± 1.69	3.49 ± 0.11	1.78 ± 0.09
Dimethyl disulphide	174 ± 5	96.6 ± 29.9	69.2 ± 11.7	65.9 ± 14.7	31.2 ± 8.6	2.93 ± 1.00
Dimethyl sulfide	1980 ± 68	79.6 ± 28.0	59.6 ± 4.7	68.7 ± 7.4	49.2 ± 6.3	24.5 ± 1.4
Dimethyl thioether	2070 ± 123	91.2 ± 38.5	72.1 ± 6.4	85.9 ± 10.9	54.4 ± 15.0	24.5 ± 1.4
Dimethyl thiosulfinate	12 ± 1	0.79 ± 0.45	1.22 ± 0.38	1.14 ± 0.21	0.93 ± 0.16	0.65 ± 0.25
Dimethyl trisulfide	5.53 ± 0.65	1.60 ± 0.34	2.15 ± 0.46	1.65 ± 1.11	1.58 ± 0.19	1.41 ± 0.30
Dipropyl sulfide	4480 ± 303	278 ± 152	148 ± 11	132 ± 33	112 ± 20	74 ± 13
Methyl mercaptan	15,300 ± 1330	4540 ± 1100	6360 ± 1120	7530 ± 1890	7920 ± 1350	2410 ± 239
Methyl propyl disulfide	1130 ± 51	114 ± 67	49.9 ± 5.2	41.3 ± 9.2	19.5 ± 5.1	2.69 ± 0.39
Pyridine	35.8 ± 3.8	1.86 ± 0.36	2.56 ± 0.19	3.27 ± 0.89	2.13 ± 0.45	0.97 ± 0.44
Trimethylpyrazine	343 ± 11	35.3 ± 19.6	16.5 ± 2.8	12.1 ± 3.3	6.12 ± 1.04	1.19 ± 0.15

**Table A6:** Fried garlic: effect of quantity of fat on the volatiles.

Volatiles	Fried Garlic (Control)	3% Fat	10% Fat	20% Fat	40% Fat	80% Fat
1,3-dithiane	115 ± 25	14.0 ± 1.8	8.16 ± 4.57	3.50 ± 0.27	2.53 ± 0.50	0.87 ± 0.39
2-ethylpyrazine	2.08 ± 1.32	0.68 ± 0.07	0.76 ± 0.64	0.45 ± 0.41	0.45 ± 0.29	0.48 ± 0.05
2-methyl-2-butenal	50.2 ± 13.0	6.88 ± 1.62	5.21 ± 1.27	3.76 ± 0.30	3.74 ± 0.77	2.03 ± 0.24
2-methylbenzaldehyde	1.82 ± 0.53	0.40 ± 0.22	0.43 ± 0.35	0.87 ± 0.37	0.63 ± 0.16	0.42 ± 0.24
2-vinyl-4H-1,3-dithiin	29.2 ± 6.7	3.25 ± 1.07	1.52 ± 0.11	1.44 ± 0.55	1.12 ± 0.45	1.71 ± 0.44
2,3-dimethylpyrazine	1.34 ± 0.79	0.76 ± 0.36	0.59 ± 0.56	0.41 ± 0.43	0.28 ± 0.17	0.34 ± 0.03
2,5-dimethylthiophene	26.8 ± 4.7	3.57 ± 0.97	2.04 ± 0.67	1.45 ± 0.08	1.98 ± 0.40	1.50 ± 0.61
Acetaldehyde	2540 ± 600	218 ± 42	272 ± 7	311 ± 21	330 ± 29	261 ± 7
Allicin	15.7 ± 9.1	8.48 ± 5.03	2.08 ± 1.18	0.97 ± 0.39	0.80 ± 0.12	0.62 ± 0.35
Allyl mercaptan	80.7 ± 9.5	43.7 ± 6.8	20.3 ± 2.7	16.7 ± 3.5	17.0 ± 3.2	11.3 ± 1.1
Allyl methyl disulfide	266 ± 39	26.6 ± 9.0	10.2 ± 4.4	3.59 ± 0.28	2.99 ± 0.23	1.00 ± 0.24
Allyl methyl sulfide	104 ± 8	30.6 ± 3.3	14.0 ± 6.7	8.63 ± 0.93	7.50 ± 1.60	7.42 ± 0.70
Allyl methyl tetrasulfide	2.42 ± 1.25	1.05 ± 0.30	0.89 ± 0.30	0.76 ± 0.25	0.49 ± 0.11	0.64 ± 0.21
Allyl methyl thiosulfinate	27.0 ± 5.1	8.94 ± 3.77	6.56 ± 0.23	6.39 ± 0.76	3.49 ± 0.63	3.36 ± 1.30
Allyl methyl trisulfide	43.5 ± 11.4	3.77 ± 1.43	2.00 ± 0.47	1.44 ± 0.56	1.02 ± 0.33	0.40 ± 0.09
Aniline	2.38 ± 0.63	1.03 ± 0.35	0.67 ± 0.09	0.52 ± 0.10	0.48 ± 0.10	0.63 ± 0.10
Diallyl disulfide	355 ± 61	20.3 ± 5.53	6.23 ± 1.40	3.42 ± 0.40	1.58 ± 0.48	0.82 ± 0.30
Diallyl sulphide	68.1 ± 10.5	12.3 ± 2.1	5.14 ± 2.59	3.21 ± 0.55	1.99 ± 0.22	1.54 ± 0.45
Diallyl tetrasulfide	2.60 ± 0.34	1.84 ± 0.84	2.35 ± 1.75	1.12 ± 0.68	1.18 ± 0.11	1.52 ± 0.40
Diallyl trisulfide	81.5 ± 19.9	8.18 ± 4.25	2.34 ± 1.62	1.02 ± 0.41	0.90 ± 0.20	1.08 ± 0.12
Dimethyl disulphide	56.5 ± 6.9	14.9 ± 0.2	5.87 ± 2.34	2.96 ± 0.89	2.12 ± 0.40	1.00 ± 0.17
Dimethyl sulfide	281 ± 46	122 ± 26	73.0 ± 26.3	50.5 ± 17.5	48.1 ± 5.8	28.8 ± 2.1
Dimethyl thioether	355 ± 50	151 ± 31	88.4 ± 33.6	56.2 ± 12.7	56.5 ± 4.1	31.5 ± 1.0
Dimethyl thiosulfinate	4.71 ± 0.77	0.94 ± 0.24	0.56 ± 0.22	0.78 ± 0.28	0.56 ± 0.20	0.70 ± 0.18
Dimethyl trisulfide	42.9 ± 16.3	6.75 ± 1.00	3.46 ± 2.21	2.67 ± 0.85	1.55 ± 0.40	1.30 ± 0.63
Dipropyl sulfide	205 ± 51	41.6 ± 11.6	21.3 ± 4.9	20.6 ± 1.2	13.0 ± 1.97	16.0 ± 2.74
Methyl mercaptan	35.0 ± 5.5	11.3 ± 2.6	6.54 ± 0.96	6.88 ± 2.35	4.70 ± 3.40	2.82 ± 3.08
Methyl propyl disulfide	36.7 ± 9.5	5.24 ± 1.09	2.78 ± 0.45	2.19 ± 0.28	0.86 ± 0.28	1.17 ± 0.17
Pyridine	2.22 ± 0.14	0.58 ± 0.18	0.47 ± 0.26	0.29 ± 0.05	0.36 ± 0.12	0.57 ± 0.24
Trimethylpyrazine	9.60 ± 1.19	1.50 ± 0.24	1.08 ± 0.32	0.78 ± 0.12	0.45 ± 0.09	0.52 ± 0.26

## References

[1] Ahmad J.I. (1996). Garlic—A panacea for health and good taste?. Nutr. Food Sci..

[2] Block E. (1985). The Chemistry of Garlic and Onions. Sci. Am..

[3] Sasi M., Kumar S., Kumar M., Thapa S., Prajapati U., Tak Y., Changan S., Saurabh V., Kumari S., Kumar A. (2021). Garlic (*Allium sativum* L.) Bioactives and Its Role in Alleviating Oral Pathologies. Antioxidants.

[4] Borlinghaus J., Albrecht F., Gruhlke M.C.H., Nwachukwu I.D., Slusarenko A.J. (2014). Allicin: Chemistry and Biological Properties. Molecules.

[5] Palazzolo C.T., Ramirez D., Locatelli D.A., Manucha W., Castro C., Camargo A. (2018). Bioaccessibility and permeability of bioactive compounds in raw and cooked garlic. J. Food Compos. Anal..

[6] Castada H.Z., Mirondo R., Sigurdson G.T., Giusti M.M., Barringer S. (2017). Deodorization of garlic odor by spearmint, peppermint, and chocolate mint leaves and rosmarinic acid. LWT.

[7] Negishi O., Negishi Y. (1999). Enzymatic Deodorization with Raw Fruits, Vegetables and Mushrooms. Food Sci. Technol. Res..

[8] Sinir G.Ö., Barrınger S. (2021). Deodorization of garlic odor by fresh and dried herbs using SIFT-MS. GIDA J. Food.

[9] Munch R., Barringer S.A. (2014). Deodorization of Garlic Breath Volatiles by Food and Food Components. J. Food Sci..

[10] Negishi O., Negishi Y., Ozawa T. (2002). Effects of Food Materials on Removal of *Allium*-Specific Volatile Sulfur Compounds. J. Agric. Food Chem..

[11] Mirondo R., Barringer S. (2016). Deodorization of Garlic Breath by Foods, and the Role of Polyphenol Oxidase and Phenolic Compounds. J. Food Sci..

[12] Hansanugrum A., Barringer S.A. (2010). Effect of Milk on the Deodorization of Malodorous Breath after Garlic Ingestion. J. Food Sci..

[13] Kühn J., Considine T., Singh H. (2006). Interactions of Milk Proteins and Volatile Flavor Compounds: Implications in the Development of Protein Foods. J. Food Sci..

[14] Druaux C., Le Thanh M., Seuvre A.M., Voilley A. (1998). Application of headspace analysis to the study of aroma compounds-lipids interactions. J. Am. Oil Chem. Soc..

[15] Ramesh C.C., Nagendra P.S. (2017). Chapter 2—An Overview of Yogurt Production and Composition. Yogurt in Health and Disease Prevention.

[16] Tamaki K., Sonoki S., Tamaki T., Ehara K. (2008). Measurement of odour after in vitro or in vivo ingestion of raw or heated garlic, using electronic nose, gas chromatography and sensory analysis. Int. J. Food Sci. Technol..

[17] Sato S., Sekine Y., Kakumu Y. (2020). Measurement of diallyl disulfide and allyl methyl sulfide emanating from human skin surface and influence of ingestion of grilled garlic. Sci. Rep..

[18] National Center for Biotechnology Information (2023). PubChem Compound Summary for CID 11617, Diallyl Sulfide. https://pubchem.ncbi.nlm.nih.gov/compound/Diallyl-sulfide.

[19] Iberl B., Winkler G., Müller B., Knobloch K. (1990). Quantitative Determination of Allicin and Alliin from Garlic by HPLC*. Planta Medica.

[20] Locatelli D.A., Altamirano J.C., González R.E., Camargo A.B. (2015). Home-cooked garlic remains a healthy food. J. Funct. Foods.

[21] Wang J., Qiao L., Wang R., Zhang N., Liu Y., Chen H., Sun J., Wang S., Zhang Y. (2023). Effect of Frying Process on the Flavor Variations of Allium Plants. Foods.

[22] Reineccius G. (2005). Flavor Chemistry and Technology.

[23] Yu T.H., Wu C.M., Ho C.T. (1993). Volatile compounds of deep-oil fried, microwave-heated and oven-baked garlic slices. J. Agric. Food Chem..

[24] Abe K., Hori Y., Myoda T. (2020). Volatile compounds of fresh and processed garlic. Exp. Ther. Med..

[25] Buttery R.G., Dante G.G., Louisa C.L. (1973). Flavor Compounds. Volatilities in Vegetable Oil and Oil-Water Mixtures. Estimation of Odor Thresholds. J. Agric. Food Chem..

[26] Alberts B., Johnson A., Lewis J. (2002). Protein Function. Molecular Biology of the Cell.

[27] Anema S.G., Li Y. (2003). Effect of pH on the Association of Denatured Whey Proteins with Casein Micelles in Heated Reconstituted Skim Milk. J. Agric. Food Chem..

[28] Mahomud M.S., Katsuno N., Nishizu T. (2017). Role of whey protein-casein complexes on yogurt texture. Rev. Agric. Sci..

[29] Vasbinder A.J., Alting A.C., Visschers R.W., de Kruif C.G. (2003). Texture of acid milk gels: Formation of disulfide cross-links during acidification. Int. Dairy J..

[30] Kühn J., Considine T., Singh H. (2008). Binding of Flavor Compounds and Whey Protein Isolate as Affected by Heat and High Pressure Treatments. J. Agric. Food Chem..

[31] Hansen A.P., Damodaran S. (1997). A Review of the Interactions between Milk Proteins and Dairy Flavor Compounds. Food Proteins and Lipids. Advances in Experimental Medicine and Biology.

[32] Eshpari H., Tong P.S., Corredig M. (2014). Changes in the physical properties, solubility, and heat stability of milk protein concentrates prepared from partially acidified milk. J. Dairy Sci..

[33] Jocelyn P.C. (1972). Chapter 16: Thioldisulfide exchange methods. Biochemistry of SH Group: The Occurrence, Chemical Properties, Metabolism and Biological Function of Thiols and Disulphides.

[34] Adams R.L., Mottram D.S., Parker J.K., Brown H.M. (2001). Flavor-protein binding: Disulfide interchange reactions between ovalbumin and volatile disulfides. J. Agric. Food Chem..

[35] Parker J.K., Mottram D.S., Adams R.L., Le Quéré J.L., Étiévant P.X. (2002). Interaction of sulphur-containing aroma compounds with proteins in both model systems and real food systems. Flavour Research at the Dawn of the Twenty-First Century: Proceedings of the 10th Weurman Flavour Research Symposium.

[36] Wiedemann C., Kumar A., Lang A., Ohlenschläger O. (2020). Cysteines and Disulfide Bonds as Structure-Forming Units: Insights from Different Domains of Life and the Potential for Characterization by NMR. Front. Chem..

[37] Whitt D.M., Pranata J., Carter B.G., Barbano D.M., Drake M.A. (2022). Effects of micellar casein concentrate purity and milk fat on sulfur/eggy flavor in ultrapasteurized milk-based beverages. J. Dairy Sci..

[38] Zhang L., Zhou R., Zhang J., Zhou P. (2021). Heat-induced denaturation and bioactivity changes of whey proteins. Int. Dairy J..

[39] Hoffman J.R., Falvo M.J. (2004). Protein—Which is Best?. J. Sports Sci. Med..

[40] Esteghlal S., Gahruie H.H., Niakousari M., Barba F.J., Bekhit A.E.-D., Mallikarjunan K., Roohinejad S. (2019). Bridging the Knowledge Gap for the Impact of Non-Thermal Processing on Proteins and Amino Acids. Foods.

[41] Aguilera J.M. (2019). The food matrix: Implications in processing, nutrition and health. Crit. Rev. Food Sci. Nutr..

